# Human reliability analysis of bronchoscope-assisted percutaneous dilatational tracheostomy: implications for simulation-based education

**DOI:** 10.1186/s41077-020-00149-7

**Published:** 2020-11-05

**Authors:** Aoife Lavelle, Mary White, Mark J.D. Griffiths, Dara Byrne, Paul O’Connor

**Affiliations:** 1 Department of Peri-operative Medicine, St Bartholomew’s Hospital, Bart’s Health NHS Trust, London, UK; 2 Irish Centre for Applied Patient Safety and Simulation, National University of Ireland, Galway, Galway, Ireland; 3 Discipline of General Practice, National University of Ireland, Galway, 1 Distillery Road, Newcastle, Galway, H91 TK33 Ireland

**Keywords:** Bronchoscope assisted percutaneous dilatational tracheostomy, Hierarchical task analysis, Human reliability analysis, Simulation

## Abstract

**Background:**

Teaching and assessing clinical procedures requires a clear delineation of the individual steps required to successfully complete the procedure. For decades, human reliability analysis (HRA) has been used to identify the steps required to complete technical procedures in higher risk industries. However, the use of HRA is uncommon in healthcare. HRA has great potential supporting simulation-based education (SBE) in two ways: (1) to support training through the identification of the steps required to complete a clinical procedure; and (2) to support assessment by providing a framework for evaluating performance of a clinical procedure. The goal of this study was to use HRA to identify the steps (and the risk associated with each of these steps) required to complete a bronchoscope-assisted percutaneous dilatational tracheostomy (BPDT). BPDT is a potentially high-risk minimally invasive procedure used to facilitate tracheostomy placement at the bedside or in the operating theatre.

**Methods:**

The subgoals, or steps, required to complete the BPDT procedure were identified using hierarchical task analysis. The Systematic Human Error Reduction and Prediction Approach (SHERPA) was then used to identify potential human errors at each subgoal, the level of risk and how these potential errors could be prevented.

**Results:**

The BPDT procedure was broken down into 395 subgoals, of which 18% were determined to be of high-risk. The most commonly identified remediation strategies for reducing the risk of the procedure included: checklist implementation and audit, statutory and mandatory training modules, simulation training, consultant involvement in all procedures, and fostering a safety-focused hospital culture.

**Conclusion:**

This study provides an approach for how to systematically identify the steps required to complete a clinical procedure for both training and assessment. An understanding of these steps is the foundation of SBE. HRA can identify ‘a correct way’ for teaching learners how to complete a technical procedure, and support teachers to give systematic and structured feedback on performance.

## Introduction

Critical care patients are among the most vulnerable patients in the hospital and, as such, are vulnerable to medical errors. Adverse event rates of 20% have been reported in the Intensive Care Unit (ICU), with 45% of these events judged to be preventable [1]. Therefore, there is great potential for reducing human error in critical care settings. In high-risk industries, such as aviation and nuclear power generation, standardisation of practice is a commonly used approach to reduce variability, and errors, in task performance. In healthcare, the learning of how to perform technical procedures is often diffuse and uneven, and based on available opportunity rather than a structured educational process [2]. Establishing a standard for task performance can be achieved through the use of human reliability analysis techniques [3]. Human reliability analysis (HRA) consists of approaches to standardise task performance, systematically identify the impact of human error on a system, and to identify ‘a correct way’ for completing a procedure. HRA techniques have great potential supporting simulation-based education (SBE) in two ways: (1) to support training through the identification of the steps required to compete a procedure; and (2) to support assessment by providing a framework for evaluating performance of a procedure.

A clear delineation of the steps required to carry out a procedure are fundamental to effective SBE approaches to learning technical procedures such as fluency training [4–6], deliberate practice [7], and mastery learning [8, 9]. However, the methods used to identify the steps in a procedure are generally not well described, nor based upon established techniques. HRA approaches have been used for decades to study human performance in high risk industries such as nuclear power generation and aviation [10]. Task analysis is a particular HRA technique that has been identified as an appropriate method of identifying the steps required to complete a procedure [11]. The information generated by a task analysis is most commonly presented hierarchically [12].

A hierarchical task analysis (HTA) is a task analysis methodology that systematically and objectively identifies and describes the actions taken to achieve a procedural objective [13]. In a HTA, the overall goal (e.g. prepare to carry out an aseptic procedure), is broken down into a series of sub-goals that must be completed to achieve the overall goal (e.g. complete hand hygiene). Although not commonly carried out in healthcare, there are examples of HTA in critical care and surgical settings including: preparing and delivering anaesthesia [14]; endotracheal suctioning [15]; ultrasound-guided right internal jugular vein cannulation [15]; rapid-sequence intubation [15]; and functional endoscopic sinus surgery [16]. However, although useful in helping to delineate the steps required to carry out a task, HTA does not allow for the identification and mitigation of potential errors in carrying out a procedure. This information on risk is important for providing feedback to learners to ensure that they take care on high-risk steps in the procedure.

Information on the level of risk associated with each step in a procedure can be generated using the Systematic Human Error Reduction and Prediction Approach (SHERPA) [17]. First used in the nuclear power industry [17], the SHERPA approach considers each subgoal of an HTA, identifies where errors can occur and offer suggestions as to how these errors can be prevented and or mitigated. This is important for teaching as it identifies the high-risk steps to the learner so that they can ensure that they take particular care to complete these steps properly.

HRA also has implication for formative and summative assessment in SBE. Task analysis has been recommended as an approach for identifying performance standards and for developing checklists for assessment in SBE [11]. However, incorporating the findings from the SHERPA, in addition to a task analysis, can greatly benefit the quality of feedback and assessment. In formative assessment the SHERPA information allows feedback to be focused on those steps that are particularly high risk, and so must be performed correctly. In summative feedback, the identification of the high-risk steps allows a grading scheme to be designed that gives a learner greater credit for performing the high risk steps in a procedure correctly as compared to the lower risk steps. Thus, a SHERPA can be used to support a more nuanced assessment of performance than an evaluation based upon a task analysis alone.

Given the importance of identifying the steps in a procedure for both teaching and assessment, the aims of this study were to (1) use HTA to provide a detailed examination of a high-risk critical care procedure—bronchoscope-assisted percutaneous dilatational tracheostomy (BPDT); (2) use SHERPA to identify those steps in the BPDT procedure that are particularly vulnerable to human error; and (3) consider the utility of carrying out these types of analyses to support SBE. It is hoped that this study will provide a ‘worked example’ of how to carry out a HTA that can be used as a model to support the development of SBE programmes to teach specific procedural skills.

## Methods

A standard approach for completing an HTA and SHERPA of a task was utilised consisting of three phases [12]: (1) Identification of the task for analysis; (2) HTA; and (3) SHERPA analysis. Each of these stages is outlined below.

### Setting

This study was carried out in the Medical Intensive Care Unit in Bart’s Heart Centre, St Bartholomew's Hospital, London. Data was collected between June and August 2019.

### Ethical approval

In May 2019, institutional approval for the study was obtained from the clinical effectiveness unit as part of a quality improvement project in St Bartholomew’s Hospital (registration number 10295). Informed consent was obtained from all participants. Consent for any video-recording and photography in the clinical environment was also obtained from the patients or their next of kin, as appropriate.

### Identification of the task for analysis

BPDT is a potentially high-risk procedure that requires simultaneous and coordinated activities of three separate physicians to achieve the desired result, i.e. a functioning tracheostomy. BPDT was chosen for analysis due to the complexity of the task, the high-risk to patients, and the paucity of educational and training aids to perform the task. BPDT is performed on patients who are predicted to have a prolonged need for mechanical ventilation, have had multiple failed trials of extubation, have copious secretions, or who are at risk of upper airway obstruction [18]. Due to the high-risk profile of patients receiving this procedure, with death occurring in 0.16% [19] to 0.25% [20] of cases, having effective training aids is imperative for both patients and trainee doctors learning the procedure.

At St Bartholomew’s Hospital, the standard approach used for the performance of percutaneous tracheostomy is a three-person technique. The airway operator ensures adequate ventilation while carefully re-positioning the endotracheal tube (ETT) to facilitate percutaneous tracheostomy insertion below it. A second operator performs video-assisted bronchoscopy to facilitate real-time visualisation of airway instrumentation during the procedure. The third operator performs the percutaneous dilatational tracheostomy. This involves the successive insertion of a cannula, guide-wire, dilators and finally tracheostomy tube into the trachea under real-time bronchoscopic visualisation. The complexity is derived from both the skills required to perform the task, and the need for precise communication between the three physicians performing the procedure and the ICU nurse assisting them.

### Hierarchical task analysis

Data for the task analysis was collected from three sources: a literature review; direct observation of subject matter experts (SMEs) performing the procedure; and interviews with SMEs. The goal of the HTA was to cover the procedure from pre-procedural safety checks to accurate confirmation of tracheostomy tube position and post-procedural care. The agreed-upon HTA was identified as a ‘correct method’ as carried out at St Bartholomew’s Hospital rather than the ‘correct method’.

#### Literature review

A literature review was performed to identify the common techniques documented in the literature for the performance of BPDT and to determine risks associated with the procedure. The review included the hospital’s local safety standards for invasive procedures (LocSSIPS) policy, relevant publications were identified using databases including PubMed, EMBASE, and the Cochrane Library. The key tasks required to complete a BPDT were extracted from the included documents. This information was reviewed by two SMEs (MG, MW) and a senior anaesthetic fellow (AL). Based upon this literature review, a provisional list of tasks, and the order in which they should be performed, was developed for each of the three roles: the airway operator; the person performing the video-assisted bronchoscopy; and the person performing the percutaneous dilatational tracheostomy.

#### Observation

The BPDT procedure was observed being performed on four patients. In total, three consultant intensivists with an anaesthesiology background, three consultant intensivists with a medical background and a senior anaesthesia/intensive care fellow were observed across the four procedures (i.e. a total of seven doctors). Each of the three roles in the BPDT was observed being performed by a different doctor in each procedure. No participant performed the same role more than once but may have participated in subsequent procedures if performing a different role. Video recordings were made of each procedure. These recordings were augmented with contemporaneous written notes.

#### Construction of HTA

A standardised approach to the development of an HTA was used [14]. The approach used to carry out the HTA is summarised below:

- 1. The general task goal was identified, i.e. a functioning tracheostomy.
- 2. The behaviours and cognitive steps required to achieve the goal were identified, i.e. the subgoals. Each subgoal was progressively decomposed until a sufficient level of detail was reached as determined by consensus between AL and MW. The level of detail was a matter of judgement and it was decided to be the point at which further decomposition was impossible or not felt to add anything substantial in terms of achieving the overall task goal [21]. To illustrate, a subgoal could be ‘infiltrate the overlying skin with local anaesthetic’, or it could be more detailed, 'insert 25G cannula just under the skin, aspirate the syringe to ensure the needle tip is not in a blood vessel, inject slowly aiming to raise a bleb of local anaesthetic causing a peau d’orange effect on skin surface'.
- 3. After describing the subgoals the next stage was to explain how the subgoals should be accomplished. This is called the plan. A plan is in the format such as ‘do X, then do Y, then do Z’, or ‘do X, or do Y and do Z’.

Based upon the literature review and the observations, draft HTAs were developed for each of the thee roles required to perform the BPTD. These draft HTAs were constructed by a senior anaesthetic fellow (AL), two consultant intensivists (MW, MG) and a human factors psychologist (POC). These HTAs were then reviewed and amended by the seven doctors who had been observed performing these roles in the observation stage described above. Any amendments that were made were discussed by AL, MW and MG until a final HTA for each role was agreed. Finally, the three individual HTAs were amalgamated into a single HTA for SHERPA analysis.

### SHERPA analysis

The subgoals of the HTA were evaluated using SHERPA analysis using a standardised approach [14, 22]. The steps in the SHERPA analysis are outlined below:

- 1. Subgoals were classified based on the behaviour required, from the following: action (e.g. aspirate nasogastric tube), information retrieval (e.g. out-rule the need for spinal precautions), checking (e.g. check for the presence of an allergy status wrist band), selection (e.g. select appropriate alarm limits for the patient) and information communication (e.g. give post tracheostomy handover to bedside nurse).
- 2. Using this behavioural classification of subgoals we determined where errors could reasonably occur and described the mode of error; as seen in Table 1.

**Table 1:** Error Classification used in SHERPA (adapted from Phipps et al. [14])

Error classification	Error mode
Action	A1—Too long/short A2—Mistimed A3—Wrong direction A4—Too little/much A5—Misaligned A6—Wrong object, right action A7—Wrong action, right object A8—Omitted A9—Incomplete A10—Wrong action and wrong object
Retrieval	R1—Information not obtained R2—Wrong information obtained R3—Information retrieval incomplete
Checking	C1—Omitted C2—Incomplete C3—Wrong object C4—Wrong check C5—Mistimed C6—Wrong check, wrong object
Selection	S1—Omitted S2—Wrong selection made
Information communication	I1—Information not communicated I2—Wrong information communicated I3—Information communication incomplete

- 3. The probability of possible errors as determined by the SMEs were described as (1); ‘low’, < 1/1000; (2) ‘medium’, > 1/1000 but < 1/100; (3) ‘high’, > 1/100 but < 1/50; and (4) ‘very high’, > 1/50 [14].
- 4. Error criticality was rated using a three-point scale: (1) low—unnoticeable clinical effect; (2) medium—transient clinical effect but not life threatening; (3) high—potentially life threatening [14].
- 5. The level of risk was determined by multiplying probability and criticality scores. A score from 0 to 2 being considered ‘low risk’, from 3 to 5 ‘medium risk’ and from 6 to 7 as ‘high-risk’.
- 6. The ‘recovery potential’ of errors was determined, i.e. could the error be identified at a later step in the HTA prior to causing adverse consequences for the patient.
- 7. Remediation strategies were suggested to reduce error frequency through the prevention of errors and mitigation of morbidity if error were to occur. These strategies were classified according to the level at which they occurred: individual, equipment, environmental and organisational.

Steps 1 to 5 of the SHERPA analysis were carried out by a senior anaesthetic fellow (AL) and a consultant intensivist (MW). Once potential errors were identified by the SHERPA analysis, AL conducted structured interviews with the primary SMEs (MW, MG) to devise strategies for prevention or remediation of potential errors. The preventative strategies identified were added to the HTA while the remediation strategies were added to the SHERPA. The SMEs reviewed the final documents with the primary author (AL) and adjusted them until the content was suitable as a familiarisation aid for trainees learning BPDT. Any differences of opinion were resolved by discussion until consensus opinion was reached. The final task analysis was then used to carry out the BPDT in a simulated setting on a manikin as an example of an application of the task analysis. This application of the task analysis was filmed.

## Results

The literature review identified four relevant documents (see Table 2). Based upon the literature review, observations and expert review, the BPDT procedure was broken down into 395 subgoals. A summary HTA for the BPDT procedure is shown in Table 3, with the complete HTA provided in Supplemental Material 1. A filmed simulated BPDT using the HTA developed in this study is available from: youtu.be/nvvLE-BimC4.

**Table 2:** References identified from literature review

Reference	Reference number
Barts NHS Trust. Local Safety Standards for Invasive Procedures based on NatSSIPs. London: Author, 2018.	[23]
Ciaglia P, Firsching R, Syniec C. Elective Percutaneous Dilatational Tracheostomy. Chest 1985;87(6):715-719.	[24]
Gadkaree SK, Schwartz D, Gerold K, Kim Y. Use of bronchoscopy in percutaneous dilational tracheostomy. JAMA Otolaryngol.. 2016,142(2):143-9.	[25]
Kost KM. Endoscopic percutaneous dilatational tracheotomy: a prospective evaluation of 500 consecutive cases. Laryngoscope. 2005;115(S107):1-30.	[26]

**Table 3:** HTA for BPDT

	Task	Plan
**1**	**Perform pre-procedural safety and documentation checks**	**All team members perform 1-2**
**1.1**	Perform hand hygiene	
**1.2**	Perform “sign in”	
**2**	**Prepare for procedure**	**All team members to perform 1; perform 2–4 concurrently; appropriate team members to perform 5-7 in order.**
**2.1**	Perform hand hygiene ± surgical scrub as appropriate	
**2.2**	Prepare drugs and IV fluids for procedure	Airway operator
**2.3**	Prepare equipment	Team to prepare equipment related to individual role
**2.4**	Use percutaneous tracheostomy equipment checklist	All team
**2.5** **2.5.1** **2.5.2** **2.5.3** **2.5.4** **2.5.5** **2.5.6** **2.5.7**	Prepare patient Increase Fi02 to 1.0 (100%) Optimise ventilation Anaesthetist patient for the procedure Tape eyes Suction oropharynx under direct vision Position patient for procedure US examination of neck	Airway operator perform 1-5
		All team members perform 6 Airway US trained team member
**2.6** **2.6.1** **2.6.2** **2.6.3** **2.6.4** **2.6.5** **2.6.6** **2.6.7**	Bronchoscopic preparation of airway Switch to catheter mount with bronchoscopic port Insert bronchoscope into trachea Suction any secretions from airway Confirm anatomy Orientate team to anatomy Withdraw ETT under bronchoscopic guidance until cuff is at/just below the level of vocal cords Confirm ability to ventilate	Airway operator and bronchoscope operator perform
**2.7**	Perform “time out”	All team
**3**	**Perform bronchoscope-assisted percutaneous dilatational tracheostomy**	**Perform in order 1-11. Perform in order 12–17 if 02 sats > 90%; if < 90% perform 13; followed by 12-17**
**3.1**	Clean skin with chlorhexidine cleaning solution × 2	
**3.2**	Apply sterile fenestrated drape to front of neck	
**3.3**	Choose tracheostomy insertion site	
**3.4**	Infiltrate overlying skin with local anaesthetic	
**3.5**	Use seeker needle to find the tracheal midline at tracheal ring 2–3^a^	
**3.6**	Make a 1–1.5 cm horizontal skin incision at this level	
**3.7**	Insert introducer cannula into trachea^a^	
**3.8**	Advance guide-wire into trachea and remove plastic cannula^a^	
**3.9**	Use mini-dilator to create tract^a^	
**3.10**	Use rhino-dilator and atraumatic introducer to dilate tract^a^	
**3.11**	Insert tracheostomy and inflate cuff^a^	
**3.12**	Perform bronchoscopy through tracheostomy to confirm position	
**3.13**	Attach ventilator to tracheostomy and confirm ventilation	
**3.14**	Secure tracheostomy: suture to skin and apply tracheostomy tube tie	
**3.15**	Remove ETT from airway	
**3.16**	Confirm tracheostomy cuff pressure is within acceptable limits	
**3.17**	Re-position patient	
**4**	**Perform follow-up care**	**All team perform in order 1–3, PT operator to perform 4–5 in any order**
**4.1**	Perform hand hygiene	
**4.2**	Confirm vital signs remain stable	
**4.3**	Perform “sign out”	
**4.4**	CXR to out-rule complications and confirm tracheostomy position	
**4.5**	Perform post-procedural documentation	

^**a**^ Interventions carried out under bronchoscopic guidance

Table 4 provides an overview of the SHERPA analysis, with the detailed analysis supplied in Supplemental Material 1. The analysis shows that the majority of subgoals were classified as action behaviours. Approximately 60% of these actions occurring during the preparation phase, and 29% occurring during the procedure. By examining the action behaviours taken during the performance of the BPDT itself, it can be seen that action behaviours account for 75% of all subgoals. Analysis of checking steps reveals that 76% of checking subgoals occur before starting the procedure. This trend is also seen with selection and information retrieval subgoals, with 83% of selection steps and 100% of information retrieval steps occurring before starting the procedure itself. Finally, looking at information communication, we find that 63% of information communication occurs during the performance of the procedure. This reflects the fact that it is a three-person technique with each team member heavily reliant on communication with the other team members.

**Table 4:** Summary of SHERPA for BPDT

		1. Safety and documentation checks	2. Preparation for procedure	3. BPDT	4. Follow-up care	Total
	**No. of subgoals**	**44**	**221**	**103**	**27**	**395**
**Type of behaviour**	Action	12	153	77	14	256 (64.8%)
	Checking	15	42	8	10	75 (19%)
	Selection	6	9	3	0	18 (4.6%)
	Retrieval	8	14	0	0	22 (5.6%)
	Information communication	3	3	15	3	24 (6%)
**Probability of error**	Error impossible	0	5	0	0	5 (1.3%)
	Low	21	56	20	6	103 (26.1%)
	Medium	7	50	36	9	102 (25.8%)
	High	10	75	38	10	133 (33.7%)
	Very high	6	35	9	2	52 (13.1%)
**Criticality of error**	Error impossible	0	5	0	0	5 (1.3%)
	Low	15	101	23	14	153 (38.7%)
	Medium	1	35	31	4	71 (18%)
	High	28	80	49	9	166 (42%)
**Risk**	Error impossible	0	5	0	0	5 (1.3%)
	Low	6	47	15	4	72 (18.2%)
	Medium	32	130	62	22	246 (62.3%)
	High	6	39	26	1	72 (18.2%)
**Remediation strategy level of intervention**^**a**^	Individual	44	186	89	27	346 (34.7%)
	Equipment	42	79	12	23	156 (15.7%)
	Environmental	42	80	7	25	154 (15.5%)
	Organisational	42	192	83	23	340 (34.1%)
	**Total**	**170**	**537**	**191**	**98**	**996**

^**a**^ Interventions can be identified at multiple levels

When looking at the probability of errors, there was a relatively even split between ‘low’ to ‘medium’ and ‘high’ to ‘very high’ risk of error (see Table 4 and Supplemental Material 1). Analysis of the criticality of errors suggested that if errors were to occur, 60% of these errors could potentially lead to clinically significant patient harm or death. Probability and criticality scores for each subgoal were multiplied, and this combined value was used to determine the level of risk posed to the patient by each subgoal. This score determined that there was a ‘medium’ level of risk associated with 62% of the subgoals; with 18% likely to pose a ‘high’ risk to patients.

Remediation strategies suggested to reduce the likelihood of error were distributed across four different levels (individual, equipment, environmental and organisational; see Table 4 and Supplemental Material 1). However, the clear majority of the remediation strategies suggested was either at the individual or organisational level. The most common remediation strategies identified included the following broad categories: checklist implementation and audit, statutory and mandatory training modules, simulation training, consultant involvement in all procedures and the encouragement of a safety-focused hospital culture.

Finally, the recovery potential from errors was assessed (see Table 4 and Supplemental Material 1). Each subgoal was reviewed in the context of the entire procedure. If a later step in the procedure could identify an error before it caused actual patient harm, it was considered to add to the recovery potential of the error. The use of checklists emerged as the most common reason for recovery potential.

## Discussion

The HTA and SHERPA methods and analyses reported in this paper provide a systematic approach to identify the steps required to complete a technical procedure. We have demonstrated that this approach can even be applied to something as complex as a BPDT. Although these HRA approaches have been used for decade to study human performance in high risk industries such as aviation and nuclear power generation, they are not widely used in healthcare [10]. It is suggested that HRA methodologies are particularly relevant to SBE such as fluency training [4–6], deliberate practice [7] and mastery learning [8, 9] which are founded upon a clear delineation of the steps required to complete the procedure.

The first stage in identifying the steps in a procedure is to conduct a task analysis. The HTA divided the BPDT procedure into 395 subgoals. Given the number of subgoals identified in our HTA, this may seem overwhelming to anyone who has not completed a HTA previously. However, depending on the intended audience and the purpose of the HTA, it may not be necessary to break a procedure down into so many subgoals [13]. For example, it may be for experienced learners that a certain level of knowledge can be assumed (e.g. how to conduct hand hygiene, how to set up a sterile field), in which case these steps do not need to be divided into more detailed subgoals. Similarly, a high level of detail may be required for the assessment of a high-stakes evaluation of performance, but not for formative assessment. Therefore, judgment and consideration is required when conducting a HTA. However, it is important to make the point that it is generally easier to make a HTA simpler and combine subgoals than the reverse. Therefore, we would recommend erring on the side of breaking the task down into slightly more subgoals than believed necessary.

The SHERPA analysis identified that more than 80% of the subgoals in the BPTD were of medium to high-risk to patients. The information on the risk of each step is very useful when learning, teaching, providing feedback or assessing someone carrying out the task as it identifies those steps in which extra care should be taken. When we looked at the checklist subgoals, we identified many steps where errors of omission or partial completion had a high probability of occurrence. It has previously been reported that only 16% of percutaneous tracheostomies have formally documented safety checks [27]. Another area of risk identified in the SHERPA analysis was the potential for communication failure at multiple points during the BPDT procedure. For example, failure to give a sufficiently detailed handover to the bedside nurse was identified as having a ‘very high’ probability of occurrence and presented a high-risk to patient safety. Therefore, it is clear that procedural adherence and communication should be a focus when teams are learning, or practicing, a BPTD in a simulated setting as well as in the actual clinical environment. It is also worth noting that the SHERPA identified issues that may not directly related to SBE such as pre-setting appropriate alarm limits and regular audit of checklist compliance. Therefore, there are implications for how to improve the safety of BPTD beyond SBE.

As with many of the other HRA reported in the healthcare literature, our HRA was clinician-led. This demonstrates that it is a feasible method for use by healthcare professionals, and support the validity of the HRA as it is being carried out by people experienced in completing the procedure [15]. The move towards a competence-based approach in graduate medical education may make HRA in healthcare more common place. A competence-based approach means that healthcare professionals will be required to demonstrate proficiency in specific technical procedures—generally in a simulated environment [28]. HRA provides a systematic method for identifying the steps in a procedure to both support learning and assessment by delineating the specifics steps required to complete a technical procedure. Given the resources required to complete an HRA it is suggested that education and simulation centres should collaborate to share HRAs to ensure that there are not duplications in effort. The HRA could be shared in written form, but ideally also in the form of a filmed simulation of the procedure being completed using the HRA (as we have done for the BPTD procedure—available from youtu.be/nvvLE-BimC4).

### Limitations

The subjectivity of the HTA and SHERPA, and the small number of SMEs from only one hospital may limit the generalisability of the findings. However, the limited number of SMEs is not just a limitation of our HRA, but is an issue with the use of HRA methodologies generally, both in healthcare [15] and other domains [29]. Due to the time required to carry out HRA, limited opportunities for observation, and the potentially limited number of suitable SMEs, it is common for there to be only a small number of participants involved in an HRA.

It is recognised that, although typical of HRA studies, the input from only a small number of SMEs is a large limitation of our analysis. Although the use a small number of subject matter experts to observe, develop, and review as part of a HRA is typical [12, 14–16], it does not mean it is desirable. It reflects the challenges of obtaining meaningful input from a sufficient number of subject matter experts. Therefore, as the HRA is based upon the opinion of a small number of SMEs, there is a need for some caution in terms of accepting the HRA reported in this paper as “a correct way” for performing a BPTD. It is suggested that the HRA reported in this paper should be regarded as a first draft, rather than a finished analysis. Just as is the case for HRAs carried out in healthcare and other high-risk work environments, these analyses are updated and change over time, and would benefit from scrutiny by a larger group of SMEs. Therefore, others should review, adapt, update and refine our HRA to reflect differences in how it is performed at different institutions, the equipment used, or the opinions of other SMEs. Our HRA, like any HRA, is not the only way to complete a BPDT, nor a finished product. Rather, the HRA is something that must be adapted to the needs and requirement of potential users.

The HRA reported in this paper provides a comprehensive description of the behaviours required to carry out a BPTD and it provides limited insights on the cognitive processes necessary to carry out the procedure. Understanding what the healthcare professionals carrying out the task were thinking about necessitates the use of cognitive task analysis techniques [30]. Cognitive task analysis has been used to study central line insertion, cricothyrotomy and the single-operator percutaneous tracheostomy technique in ICU [31, 32]. Future research should also consider the cognitive processes of the healthcare professional as they carry out the procedure.

## Conclusion

This study has demonstrated that HRA provides an approach to systematically identify the steps required to perform a technical procedure. An understanding of these steps is the foundation of effective SBE methods to teaching technical procedures. HRA can provide an approach to identify ‘a correct way’ for teaching learners how to complete a technical procedure, support structured feedback, and benefit performance assessment.

## Supplementary Information

**Additional file 1.**

## Data Availability

All collected data is available in Supplemental Materials 1.

## References

[1] Rothschild JM, Landrigan CP, Cronin JW, Kaushal R, Lockley SW, Burdick E, Stone PH, Lilly CM, Katz JT, Czeisler CA, Bates DW (2005). The critical care safety study: The incidence and nature of adverse events and serious medical errors in intensive care. Crit Care Med..

[2] Grantcharov TP, Reznick RK (2008). Teaching procedural skills. BMJ.

[3] Kirwan B (1998). Human error identification techniques for risk assessment of high-risk systems- Part 1: review and evaluation of techniques. App Erg..

[4] Lydon S, Burns N, Healy O, O'Connor P, Reid-McDermott B, Byrne D (2017). Preliminary evaluation of the efficacy of an intervention incorporating precision teaching to train procedural skills among final cycle medical students. BMJ STEL.

[5] Lydon S, McDermott BR, Ryan E, O’Connor P, Dempsey S, Walsh C, Byrne D (2019). Can simulation-based education and precision teaching improve paediatric trainees’ behavioural fluency in performing lumbar puncture? A pilot study. BMC Med Ed.

[6] Reid-McDermott B, Browne M, Byrne D, O’Connor P, O’Dowd E, Walsh C, Madden C, Lydon S (2019). Using simulation to explore the impact of device design on the learning and performance of peripheral intravenous cannulation. Advanc Sim..

[7] Ericsson KA (2004). Deliberate practice and the acquisition and maintenance of expert performance in medicine and related domains. Acad Med..

[8] McGaghie W, Siddall V, Mazmanian P, Myers J, Committee AC of CPH and SP (2009). American College of Chest Physicians Evidence-Based Educational Guidelines. Chest..

[9] Barsuk J, McGaghie WC, Cohen ER, O’Leary KJ, Wayne DB (2009). Simulation-based mastery learning reduces complications during central venous catheter insertion in a medical intensive care unit. Crit Care Med..

[10] Sujan MA, Embrey D, Huang H (2020). On the application of human reliability analysis in healthcare: opportunities and challenges. Reliab Eng Syst Saf.

[11] Lammers RL, Davenport M, Korley F, Griswold-Theodorson S, Fitch MT, Narang AT, Evans LV, Gross A, Rodriguez E, Dodge KL, Hamann CJ (2008). Teaching and assessing procedural skills using simulation: metrics and methodology. Acad Emerg Med..

[12] Lane R, Stanton N, Harrison D (2006). Applying hierarchical task analysis to medication administration errors. Appl Erg..

[13] Kirwan B, Ainsworth LK, editors. A guide to task analysis: the task analysis working group. Boca Raton: CRC press; 1992.

[14] Phipps D, Meakin GH, Beatty PC, Nsoedo C, Parker D (2008). Human factors in anaesthetic practice: insights from a task analysis. Brit J Anaesth.

[15] Reddy K, Byrne D, Breen D, Lydon S, O’Connor P. The application of human reliability analysis to three critical care procedures. Rel Eng System Saf. 2020;203(11):107–16.

[16] Corbett M, O'Connor P, Byrne D, Thornton M, Keogh I (2019). Identifying and reducing risks in functional endoscopic sinus surgery through a hierarchical task analysis. Laryngoscope Investig Otolaryngol..

[17] Embery DE. SHERPA: a systematic human error reduction and prediction approach. Paper presented at the International Topical Meeting on Advances in Human Factors in Nuclear Power systems, Knoxville Tennessee. 1986.

[18] De Leyn P, Bedert L, Delcroix M, Depuydt P, Lauwers G, Sokolov Y, Van Meerhaeghe A, Van Schil P (2007). Tracheotomy: clinical review and guidelines. Euro J Card Thor Surg..

[19] Dennis B, Eckert M, Gunter O, Morris J, May A (2013). Safety of bedside percutaneous tracheostomy in the critically ill: evaluation of more than 3,000 procedures. J Amer Coll Surg..

[20] McCormick B, Manara A (2005). Mortality from percutaneous dilatational tracheostomy. A report of three cases. Anaesth..

[21] Shepherd A (2001). Hierarchical Task Analysis.

[22] Stanton N, Salmon P, Walker G, Baber C, Jenkins D (2005). Human factors methods.

[23] Barts NHS Trust (2018). Local Safety Standards for Invasive Procedures based on NatSSIPs.

[24] Ciaglia P, Firsching R, Syniec C (1985). Elective Percutaneous Dilatational Tracheostomy. Chest.

[25] Gadkaree SK, Schwartz D, Gerold K, Kim Y (2016). Use of bronchoscopy in percutaneous dilational tracheostomy. JAMA Otolaryngol.

[26] Kost KM (2005). Endoscopic percutaneous dilatational tracheotomy: a prospective evaluation of 500 consecutive cases. Laryngoscope.

[27] Wilkinson K, Freeth H, Martin I (2015). Are we ‘on the right trach?’ The National Confidential Enquiry into Patient Outcome and Death examines tracheostomy care. J Laryngol Otol.

[28] Iobst WF, Sherbino J, Cate OT, Richardson DL, Dath D, Swing SR, Harris P, Mungroo R, Holmboe ES, Frank JR (2010). Competency-based medical education in postgraduate medical education. Med Teach.

[29] Stanton NA, Stevenage SV (1998). Learning to predict human error: issues of acceptability, reliability and validity. Ergo.

[30] Seamster TL, Redding RE, Kaempf GL (1997). Applied cognitive task analysis in aviation.

[31] Sullivan M, Brown C, Peyre S, Salim A, Martin M, Towfigh S, Grunwald T (2007). The use of cognitive task analysis to improve the learning of percutaneous tracheostomy placement. Amer J Surg.

[32] Yates K, Sullivan M, Clark R (2012). Integrated studies on the use of cognitive task analysis to capture surgical expertise for central venous catheter placement and open cricothyrotomy. Amer J Surg.

